# Phytohormone Response to Exogenous Nitric Oxide in Cucumber Under Low-Temperature Stress

**DOI:** 10.3390/plants14213275

**Published:** 2025-10-27

**Authors:** Pei Wu, Zhifeng Yang, Qiusheng Kong, Huimei Cui, Yumei Liu, Rongrong Dong, Caixia Zheng, Huiying Liu, Jinxia Cui

**Affiliations:** 1 College of Agricultural and Biological Engineering, Heze University, Heze 274000, China; peiwu0622@163.com (P.W.);; 2 Key Laboratory of Special Fruits and Vegetables Cultivation Physiology and Germplasm Resources Utilization of Xinjiang Production and Construction Crops, Department of Horticulture, Agricultural College, Shihezi University, Shihezi 832003, Chinachm_agr@shzu.edu.cn (H.C.); 3 College of Horticulture and Forestry Sciences, Huazhong Agricultural University, Wuhan 430070, China

**Keywords:** cucumber, low temperature, phytohormone, flavonoid, glutathione biosynthesis

## Abstract

To elucidate the comprehensive mechanism by which nitric oxide (NO) enhances low-temperature tolerance in cucumber, we utilized two cucumber cultivars (Jinyan No. 4 and Jinyou No. 1) as experimental materials. By integrating transcriptomic analysis with physiological indicators, we investigated the physiological and molecular mechanisms underlying the NO-mediated improvement of cold tolerance. Both molecular and physiological data revealed that phytohormone signal transduction and alpha-linolenic acid metabolism were significantly affected by low-temperature stress alone and in combination with exogenous SNP treatment in both cultivars. Under low-temperature stress, most transcripts associated with abscisic acid (ABA) biosynthesis, ABA signal transduction, and flavonoid biosynthesis were coordinately downregulated in cucumber. In contrast, transcripts related to secondary metabolism, lipid metabolism, glutathione biosynthesis, and hormone signal transduction—including salicylic acid (SA), ethylene (ETH), gibberellin (GA), and jasmonic acid (JA) pathways—were coordinately upregulated. Additionally, exogenous SNP was found to regulate both phytohormone signal transduction and endogenous hormone levels. These results suggest that exogenous NO improves low-temperature tolerance in cucumber seedlings primarily by modulating phytohormone signaling and secondary metabolism.

## 1. Introduction

Cucumber (*Cucumis sativus* L.), originating in tropical and subtropical regions, is an economically important vegetable crop worldwide. Global cucumber production has increased steadily since 1998, reaching approximately 80 million metric tons by 2016. The crop is rich in bioactive compounds such as cucurbitacin C, along with trace amounts of vitamins E and B1. These health-promoting properties, combined with their economic value, have contributed to their widespread cultivation in China [1]. As the world’s leading producer of cucumber, China benefits from intensive cultivation practices, particularly in its northern regions, where the climate is highly suitable for cucumber growth. However, as a summer crop, cucumber is highly sensitive to low temperatures.

The optimal growth temperature for cucumber ranges from 18 °C and 25 °C. Temperatures exceeding 30 °C or falling below 12 °C can significantly inhibit growth [2]. In early spring and during off-season cultivation in solar greenhouses, low temperatures pose a serious challenge for many horticultural crops, including cucumber [3,4]. Chilling stress is a major factor leading to reduced growth and yield. Previous studies have indicated that low temperature impairs the growth and development of cucumber seedlings by decreasing enzyme activity, increasing reactive oxygen species (ROS) accumulation, destabilizing membranes, reducing chlorophyll (Chl) biosynthesis, and impairing photosynthetic machinery [5]. Additionally, low temperature disrupts the photosynthetic system and carbohydrate metabolism in cucumber [6,7]. Compared with other crops, the understanding of responsive and tolerance mechanisms of cucumber to low temperature stress remains very limited, which hinders efforts to improve its chilling tolerance. Therefore, investigating the underlying mechanisms of cucumber under low-temperature stress is of great importance.

Adaptation to low temperatures is a dynamic stress-response process involving complex interactions across multiple regulatory levels. The antioxidant system serves as the first line of defense against low-temperature stress in cucumber [6]. Transcriptome analyses have revealed that transcription factors such as *WRKY*, *ICE*, and *CBF* are involved in low-temperature response [8,9,10,11]. Furthermore, physiological and biochemical studies have shown that endogenous hormones, including auxin (IAA), abscisic acid (ABA), jasmonic acid (JA), zeatin [12], and gibberellin (GA), also participate in the response to low-temperature stress [13]. These findings illustrate how phytohormone profiles in cucumber shift under low-temperature conditions. However, the molecular mechanisms by which endogenous hormones mediate cucumber adaptation to low-temperature stress require further investigation.

Although plants have evolved a range of mechanisms to adapt to low-temperature stress, studying the role of signaling molecules such as nitric oxide (NO) in these adaptive processes remains highly valuable. Such investigations can provide deeper insights into plant resistance mechanisms. As a multifunctional signaling molecule, NO has attracted considerable research interest in plant biology. Previous studies have reported that NO is involved in various biochemical and physiological processes under low-temperature stress [6,7,14,15,16]. Moreover, NO interacts with phytohormones to regulate plant growth, development, and abiotic stress tolerance. For instance, NO has been shown to participate in salicylic acid (SA)-enhanced cell wall phosphorus remobilization in rice [17]. Esim and Atici reported that the combined treatment of NO and SA alleviated chilling stress-induced oxidative damage in wheat [18]. Additionally, NO was implicated in mitigating the GA-induced programmed cell death (PCD) of drought-stressed rice aleurone layers [19]. Although these studies indicated crosstalk between NO and phytohormone in plant growth and stress responses, the molecular mechanism by which NO regulates phytohormone synthesis and signaling warrant further investigation. 

Based on our previous research, we found that NO regulates photosynthesis, lignin synthesis, phytohormone signal transduction, phenylalanine metabolism, the cell cycle, and GA synthesis by modulating the expression of key transcription factors and their downstream genes [16]. In this study, two cucumber cultivars were selected to examine their responses to low-temperature stress through RNA sequencing, along with measurements of phytohormone levels and antioxidant content. We identified differentially expressed genes (DEGs), changes in the content of phytohormones (SA, JA, GA, and ABA) and antioxidants (GSH and flavonoids), and shifts in the relative content of unsaturated fatty acids. These analyses help elucidate the transcriptional and physiological changes regulated by low-temperature stress and NO. The findings are expected to enhance our understanding of cucumber responses to low-temperature stress and the role of NO in this process.

## 2. Results

Phytohormones serve as key signals in activating plant resistance. When exposed to adversity stress, plants can rapidly and precisely initiate the expression of relevant genes to cope with abiotic stress. Given the significant enrichment of DEGs in pathways, including plant hormone signal transduction, alpha-linolenic acid metabolism, phenylpropanoid biosynthesis, linoleic acid metabolism, and diterpenoid biosynthesis pathways in cucumber seedlings (Figures S3–S5), we further compared the expression patterns of genes involved in these pathways of the two cucumber cultivars under low-temperature stress and exogenous NO treatment.

### 2.1. Expression Patterns of DEGs Enriched in SA Signal Transduction

**Figure 1:**
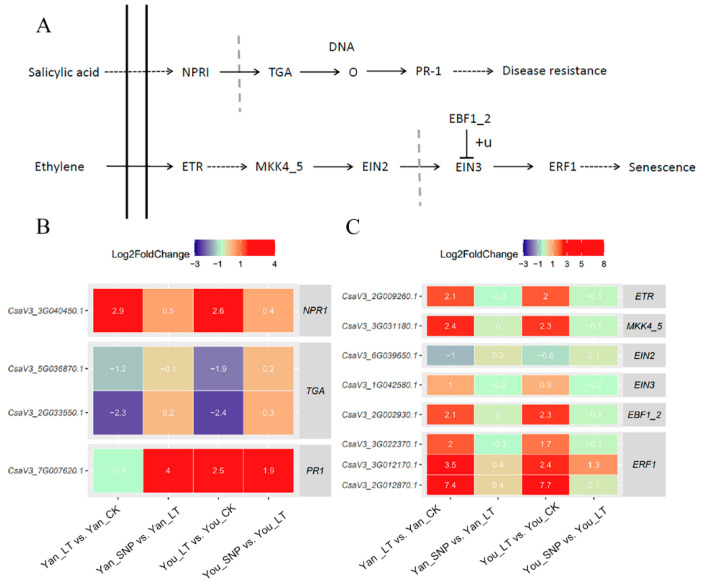
Expression patterns of differentially expressed genes (DEGs) involved in the ETH and SA signal transduction pathways. (**A**) Simplified ETH and SA signal transduction pathways; (**B**) expression patterns of transcripts involved in the salicylic acid signal transduction pathways; (**C**) expression patterns of transcripts involved in the ETH signal transduction pathways. The treatment groups were defined as follows: Yan_CK and You_CK: the seedlings of ‘Jinyan No 4’ and Jinyou No. 1 were pretreated with distilled water once daily for two days and then grown under normal conditions for 24 h, respectively; Yan_LT and You_LT: the seedlings of ‘Jinyan No 4’ and Jinyou No. 1 were pretreated similarly with distilled water but then exposed to low temperature for 24 h, respectively; Yan_SNP and You_SNP: the seedlings of ‘Jinyan No 4’ and Jinyou No. 1 were pretreated with exogenous SNP (200 μmol · L^−1^) once daily for two days, then grown under low temperature for 24 h, respectively.

NPR1 (non-expressor of PR1) mediates the expression of PR1 (pathogenesis-related protein 1) by interacting with TGA (TGACG-binding factor 1) transcription factors, thereby activating SA-dependent defense responses. In this study, we identified three TGA genes, one NPR1 gene, and one PR1 gene within the SA signaling pathway. Under low-temperature stress, *NPR1* was upregulated in both ‘Jinyan No. 4’ (Yan) and ‘Jinyou No. 1’ (You) cultivars. In contrast, two *TGA* genes were downregulated by low-temperature but induced by exogenous SNP. Meanwhile, *PR1* expression was significantly upregulated by low temperature in ‘You’ but not in ‘Yan’. However, exogenous SNP application substantially upregulated *PR1* in both cultivars compared to their low-temperature-treated counterparts (Figure 1B). These results suggest that low-temperature stress activates the SA pathway primarily through *NPR1* and, in a cultivar-specific manner, *PR1*. Furthermore, exogenous NO can enhance this defense response by upregulating *PR1* expression in both cucumber cultivars.

### 2.2. Expression Patterns of DEGs Enriched in ETH Signal Transduction

ETH binding to its receptor (ETR) inactivates the negative regulator CTR1 (constitutive triple response 1). This inactivation prevents CTR1 from phosphorylating EIN2 (ethylene-insensitive protein 2), leading to EIN2 activation. The C-terminal end of EIN2 (EIN2 CEND) is then cleaved and translocated into the nucleus, while it promotes EIN3/EIL1 accumulation by inhibiting their ubiquitination and degradation mediated by EBF1 _2 (EIN3-binding F-Box 1_2). Subsequently, EIN3/EIL1 activates the transcription of ERF1 (ethylene-responsive factor1) and other downstream target genes [20]. In this study, we identified genes encoding *ETR*, *MKK4_5*, *EIN3*, *EIN2*, *EBF1_2*, and *ERF1* in the ETH signal pathway. As shown in Figure 1C, *ETR*, *MKK4_5*, *EBF1_2*, and three *ERF1* genes were significantly upregulated under low-temperature stress in both cultivars, while *EIN2* and *EIN3* expression remained unchanged. Most ETH pathway genes showed no differential expression in response to exogenous SNP, except for one *ERF1* gene. These findings suggest that low-temperature stress activates ETH receptors (*ETR*) in both cucumber cultivars, initiating a signaling cascade through MKK4_5, EIN2, EIN3, and EBF1_2 that ultimately induces downstream *ERF1* genes within the ETH signaling pathway.

### 2.3. Expression Patterns of DEGs Enriched in JA and Methyl Jasmonate (MeJA) Synthesis, and the Signal Transduction Pathways of JA

**Figure 2:**
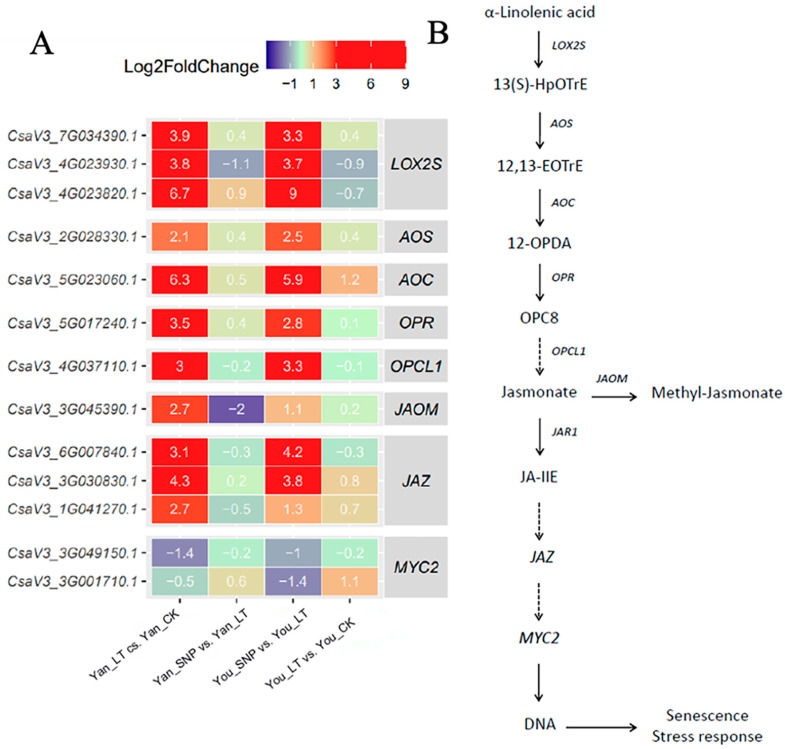
Expression patterns of differentially expressed genes (DEGs) involved in the JA and jasmonate (MeJA) biosynthesis and JA signal transduction pathways. (**A**) Expression patterns of transcripts involved in the JA and MeJA biosynthesis and JA transduction pathways; (**B**) simplified JA biosynthesis and JA signal transduction pathways. The treatment groups were defined as follows: Yan_CK and You_CK: the seedlings of ‘Jinyan No 4’ and Jinyou No. 1 were pretreated with distilled water once daily for two days and then grown under normal conditions for 24 h, respectively; Yan_LT and You_LT: the seedlings of ‘Jinyan No 4’ and Jinyou No. 1 were pretreated similarly with distilled water but then exposed to low temperature for 24 h, respectively; Yan_SNP and You_SNP: the seedlings of ‘Jinyan No 4’ and Jinyou No. 1 were pretreated with exogenous SNP (200 μmol · L^−1^) once daily for two days, then grown under low temperature for 24 h, respectively.

The alpha-linolenic acid metabolic pathway culminates in the synthesis of JA and MeJA, with JA converted to MeJA by JAOM (jasmonate oxygen methyltransferase). As shown in Figure 2, we identified in cucumber seedlings three *LOX2S* transcripts and one *AOS*, one *AOC*, one *OPR*, one *OPCL1*, and one *JAOM* gene involved in this pathway. Under low-temperature stress, the expression of these genes was significantly upregulated in both cucumber cultivars. However, one *LOX2S* (*CsaV3_4G023930.1*) gene and *JAOM* were significantly downregulated in Yan_SNP vs. Yan_LT, but not in You_SNP vs. You_LT (Figure 2). The results indicate that low temperature induces the expression of *LOX2S*, *AOS*, *AOC*, *OPR*, *OPCL1*, and *JAOM* in the alpha-linolenic acid pathway, while exogenous SNP appears to participate partially in this process.

In the JA signaling pathway, three transcripts encoding the negative regulator *JAZ* were significantly upregulated in both cultivars under low temperature, but showed no significant change in response to exogenous SNP (Figure 2). Meanwhile, two *MYC2* transcripts were downregulated by low temperature. Notably, one *MYC2* gene was significantly downregulated in You_SNP vs. You_LT, but not in Yan_SNP vs. Yan_LT (Figure 2). These findings suggest that low temperature activates the JAZ protein, thereby suppressing *MYC2* expression in cucumber seedlings. Exogenous SNP, however, appears to inhibit *JAZ* expression and subsequently alleviate its repression on *MYC2* (Figure 2A), ultimately activating the JA signaling pathway in ‘Jinyou No. 1’.

### 2.4. Expression Patterns of DEGs Enriched in Gibberellin (GA) Biosynthesis and Signal Transduction

**Figure 3:**
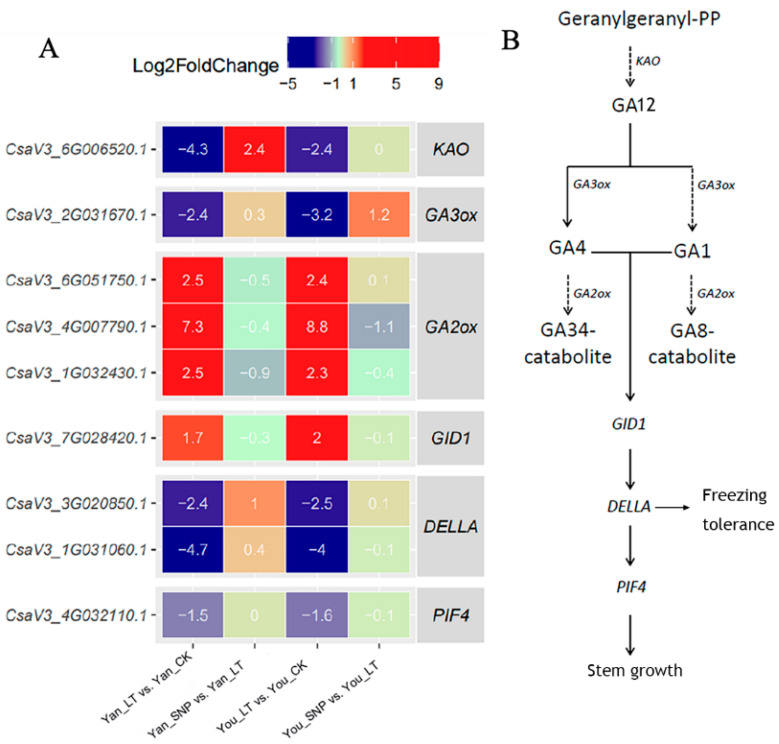
Expression patterns of differentially expressed genes (DEGs) involved in the gibberellin biosynthesis and gibberellin signal transduction pathways. (**A**) Expression patterns of genes involved in the gibberellin biosynthesis and gibberellin signal transduction pathways; (**B**) a simplified gibberellin biosynthesis and signal transduction pathway. The treatment groups were defined as follows: Yan_CK and You_CK: the seedlings of ‘Jinyan No 4’ and Jinyou No. 1 were pretreated with distilled water once daily for two days and then grown under normal conditions for 24 h, respectively; Yan_LT and You_LT: the seedlings of ‘Jinyan No 4’ and Jinyou No. 1 were pretreated similarly with distilled water but then exposed to low temperature for 24 h, respectively; Yan_SNP and You_SNP: the seedlings of ‘Jinyan No 4’ and Jinyou No. 1 were pretreated with exogenous SNP (200 μmol · L^−1^) once daily for two days, then grown under low temperature for 24 h, respectively.

The biosynthesis of gibberellin (GA) in higher plants occurs in three stages. In the second stage, GA12 serves as the initial GA product and is catalyzed by *GA3ox* to form bioactive gibberellins GA1 and GA4. Meanwhile, *GA2ox* (2-oxoglutarate-dependent dioxygenase) degrades bioactive GAs [21]. In the diterpenoid biosynthesis pathway, we identified five genes: one encoding *KAO*, one encoding *GA3ox*, and three encoding *GA2ox* (Figure 3). Under low-temperature stress, *KAO* and *GA3ox* were significantly downregulated, whereas *GA2ox* genes were upregulated in both cultivars. Following exogenous SNP treatment, *KAO*, *GA3ox*, and one *GA2ox* gene exhibited differential expression patterns between cultivars: *KAO* was upregulated in ‘Yan’, while *GA3ox* was upregulated in ‘You’. Most *GA2ox* genes were downregulated in both cultivars under SNP treatment, though one (*CsaV3_4G007790.1*) was significantly downregulated only in ‘You’ (Figure 3).

DELLA proteins act as negative regulators in GA signaling. GA binding to GID1 (GA-INSENSITIVE DWARF 1) promotes GID1-DELLA interaction, leading to DELLA ubiquitination and degradation via the 26S proteasome. DELLA also interacts with transcription regulators to modulate downstream gene expression. In this study, one *GID1* gene and two *DELLA* genes were identified. Low temperature significantly upregulated *GID1* and downregulated *DELLA* in both cultivars (Figure 3). Exogenous SNP partially alleviated the low-temperature effect, though not significantly. These results indicate that under low-temperature stress, *DELLA* repression and *GID1* activation maintain GA signaling activity. Under combined SNP and low-temperature treatment, *GID1* activation was suppressed, *DELLA* inhibition was attenuated, and downstream *PIF4* expression was inhibited.

### 2.5. Expression Patterns of DEGs Enriched in ABA Biosynthesis and Signal Transduction Pathway

**Figure 4:**
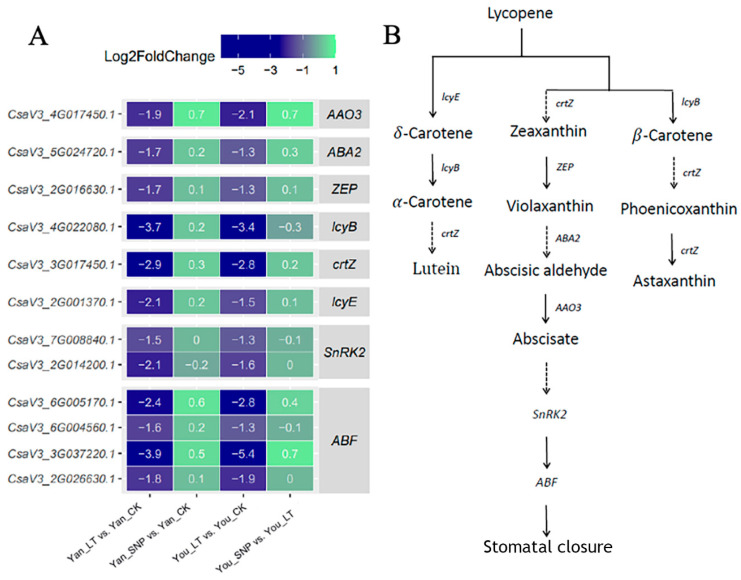
Expression patterns of differentially expressed genes involved in the abscisic acid biosynthesis and abscisic acid signal transduction pathways. (**A**) Expression patterns of transcripts involved in the abscisic acid biosynthesis and abscisic acid signal transduction pathways; (**B**) simplified abscisic acid biosynthesis and abscisic acid signal transduction pathways. The treatment groups were defined as follows: Yan_CK and You_CK: the seedlings of ‘Jinyan No 4’ and Jinyou No. 1 were pretreated with distilled water once daily for two days and then grown under normal conditions for 24 h, respectively; Yan_LT and You_LT: the seedlings of ‘Jinyan No 4’ and Jinyou No. 1 were pretreated similarly with distilled water but then exposed to low temperature for 24 h, respectively; Yan_SNP and You_SNP: the seedlings of ‘Jinyan No 4’ and Jinyou No. 1 were pretreated with exogenous SNP (200 μmol · L^−1^) once daily for two days, then grown under low temperature for 24 h, respectively.

SNRK2 (SNF1-related protein kinase 2), a positive regulator in the ABA signaling pathway, activates ABF (ABA-responsive element (ABRE)-binding transcription factor) to induce ABA-responsive gene expression. In this study, two *SNRK2* genes and four *ABF* genes were significantly downregulated under low-temperature stress in both cucumber cultivars. However, the expression of these genes showed no significant changes in response to exogenous SNP treatment (Figure 4). Additionally, genes involved in ABA biosynthesis also exhibited a downregulation trend under low-temperature stress.

### 2.6. Expression Patterns of DEGs Were Involved in Flavonoid, and Glutathione (GSH) Metabolism Pathways

**Figure 5:**
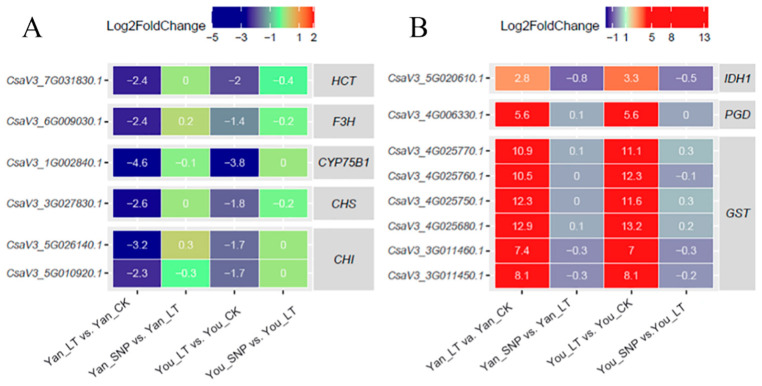
Expression patterns of DEGs involved in flavonoid metabolism (**A**) and glutathione metabolism (**B**). The treatment groups were defined as follows: Yan_CK and You_CK: the seedlings of ‘Jinyan No 4’ and Jinyou No. 1 were pretreated with distilled water once daily for two days and then grown under normal conditions for 24 h, respectively; Yan_LT and You_LT: the seedlings of ‘Jinyan No 4’ and Jinyou No. 1 were pretreated similarly with distilled water but then exposed to low temperature for 24 h, respectively; Yan_SNP and You_SNP: the seedlings of ‘Jinyan No 4’ and Jinyou No. 1 were pretreated with exogenous SNP (200 μmol · L^−1^) once daily for two days, then grown under low temperature for 24 h, respectively.

All identified genes in the flavonoid biosynthesis pathway, including *HCT*, *F3H*, *CYP75B1*, *CHS*, and *CHI*, were downregulated under low-temperature treatment stress (Figure 5A). However, exogenous SNP alleviates this repression of flavonoid synthesis-related genes.

As a key antioxidant in the non-enzymatic system, GSH can be conjugated with peroxides via GST (glutathione S-transferase), thereby reducing cellular oxidative damage. A total of six *GST* genes, one IDH1, and one PGD gene were identified in cucumber seedlings (Figure 5B). All of these genes were upregulated under low-temperature stress in both cultivars. In contrast, exogenous SNP attenuated the induction of *GST* genes at low temperatures (Figure 5B).

### 2.7. The Effect of Exogenous SNP on Phytohormone Content in Cucumber Leaves Under Low Temperature Stress

**Figure 6:**
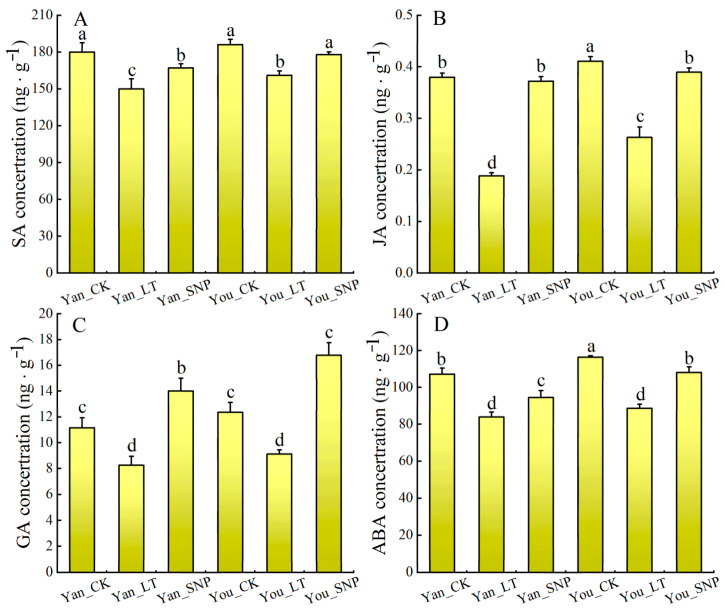
The contents of SA (**A**), JA (**B**), GA (**C**), and ABA (**D**) in each treatment of cucumber seedlings. The treatment groups were defined as follows: Yan_CK and You_CK: the seedlings of ‘Jinyan No 4’ and Jinyou No. 1 were pretreated with distilled water once daily for two days and then grown under normal conditions for 24 h, respectively; Yan_LT and You_LT: the seedlings of ‘Jinyan No 4’ and Jinyou No. 1 were pretreated similarly with distilled water but then exposed to low temperature for 24 h, respectively; Yan_SNP and You_SNP: the seedlings of ‘Jinyan No 4’ and Jinyou No. 1 were pretreated with exogenous SNP (200 μmol · L ^−1^) once daily for two days, then grown under low temperature for 24 h, respectively. Lowercase letters indicate significant differences at *p* < 0.05.

As shown in Figure 6, low temperature significantly reduced the contents of SA, JA, GA, and ABA in cucumber leaves compared to the control (Yan_CK and You_CK). In contrast, exogenous SNP applied under low-temperature stress (Yan_SNP and You_SNP) significantly increased the levels of these phytohormones. Notably, elevated NO levels partially restored SA, JA, and ABA to near-normal levels, while GA content was markedly enhanced beyond the control condition.

### 2.8. The Effect of Exogenous NO on Antioxidant Substances and Linoleic Acid Content in Cucumber Leaves Under Low Temperature Stress

**Figure 7:**
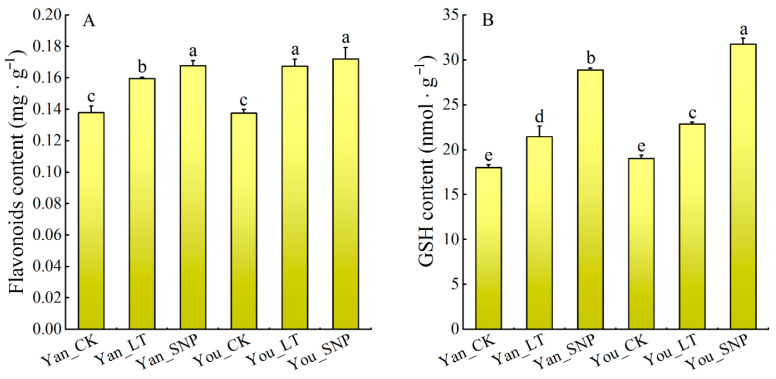
The contents of flavonoids (**A**) and GSH (**B**) in each treatment of cucumber seedling leaves. The treatment groups were defined as follows: Yan_CK and You_CK: the seedlings of ‘Jinyan No 4’ and Jinyou No. 1 were pretreated with distilled water once daily for two days and then grown under normal conditions for 24 h, respectively; Yan_LT and You_LT: the seedlings of ‘Jinyan No 4’ and Jinyou No. 1 were pretreated similarly with distilled water but then exposed to low temperature for 24 h, respectively; Yan_SNP and You_SNP: the seedlings of ‘Jinyan No 4’ and Jinyou No. 1 were pretreated with exogenous SNP (200 μmol · L^−1^) once daily for two days, then grown under low temperature for 24 h, respectively. Lowercase letters indicate significant differences at *p* < 0.05.

Compared to the control (Yan_CK and You_CK), low-temperature stress (Yan_LT and You_LT) significantly increased the contents of GSH and flavonoids in the cucumber leaves. Furthermore, the combined treatment of low-temperature and exogenous SNP (Yan_SNP and You_SNP) led to a further significant increase in both GSH and flavonoid levels compared to low-temperature treatment alone (Figure 7).

### 2.9. Validation of Transcriptomic Data Accuracy by qRT-PCR

**Figure 8:**
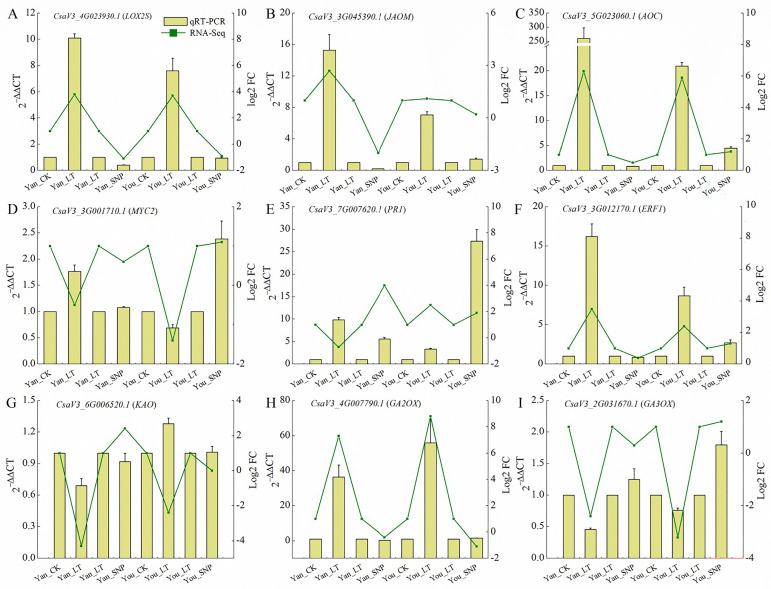
The relative expression levels of the selected genes were measured using RT-qPCR. (**A**) the expression level of *LOX2S*; (**B**) the expression level of *JAOM*; (**C**) the expression level of *AOC*; (**D**) the expression level of *MYC2*; (**E**) the expression level of *PR1*; (**F**) the expression level of *ERF1*; (**G**) the expression level of *KAO*; (**H**) the expression level of *GA2ox*; (**I**) the expression level of *GA3ox*. The expression of all nine genes in Yan_CK and You_CK was used as the baseline to normalize their expression under Yan_LT and You_LT treatments, respectively. Similarly, the expression levels in Yan_LT and You_LT were normalized (resulting in Yan_LT-M and You_LT-M) to assess the expression in Yan_SNP and You_SNP, respectively. The treatment groups were defined as follows: Yan_CK and You_CK: the seedlings of ‘Jinyan No 4’ and Jinyou No. 1 were pretreated with distilled water once daily for two days and then grown under normal conditions for 24 h, respectively; Yan_LT and You_LT: the seedlings of ‘Jinyan No 4’ and Jinyou No. 1 were pretreated similarly with distilled water but then exposed to low temperature for 24 h, respectively; Yan_SNP and You_SNP: the seedlings of ‘Jinyan No 4’ and Jinyou No. 1 were pretreated with exogenous SNP (200 μmol · L^−1^) once daily for two days, then grown under low temperature for 24 h, respectively.

Nine DEGs were selected for qRT-PCR validation (Figure 8), including four involved in JA and MeJA synthesis and signaling (Figure 8A–D), one related to SA regulation (Figure 8E), one associated with ETH signaling (Figure 8F), and three involved in GA synthesis and metabolism (Figure 8G–I). Compared with Yan_CK, the expressed levels of *LOS2S* (*CsaV3_4G023930.1*), *JAOM* (*CsaV3_3G045390.1*), *AOC* (*CsaV3_5G023060.1*), *PR1* (*CsaV3_7G007620.1*), *ERFI* (*CsaV3_3G012170.1*), and *GA2ox* (*CsaV3_4G007790.1*) were upregulated in Yan_LT. A similar expression pattern was observed in You_LT relative to You_CK. In contrast, *GA3ox* (*CsaV3_2G031670.1*) was downregulated in both cultivars under low-temperature stress. Under combined low-temperature and exogenous NO treatment (Yan_SNP and You_SNP), *LOX2S* was downregulated, while *MYC2*, *PR1*, and *GA3ox* were upregulated compared to low-temperature treatment alone. Notably, *JAOM* and *ACO* were downregulated in Yan_SNP vs. You_LT, but upregulated in You_SNP vs. You_LT. These qRT-PCR results are consistent with the RNA-Seq data, confirming the reliability of the transcriptome expression profiles.

## 3. Discussion

Due to frequent climatic changes, temperature-related challenges have become a major concern in plant science worldwide. Low-temperature stress is a key environmental constraint that adversely affects plant growth, development, and metabolism and, in severe cases, leads to plant death. Plants adapt to low-temperature stress through alterations in membrane composition, activation of reactive oxygen species scavenging systems, secondary metabolism, cytochrome P450, and plant hormone signaling pathways [22,23]. Consistent with previous studies, we found that genes responsive to exogenous SNP under low-temperature stress were enriched in pathways related to plant hormone signal transduction pathways, secondary metabolism, and metabolism of xenobiotics by cytochrome P450.

Numerous studies have shown that nitric oxide (NO) plays a critical role in mediating plant responses to various stresses. It regulates protein kinase and antioxidant enzyme activities, mobilizes flavonoids and other secondary metabolites such as SA, ET, IAA, ABA, and JA, and activates genes encoding ABC transporter and GSTs, thereby influencing plant growth [6,16,24,25]. KEGG enrichment analysis in this study further revealed that exogenous SNP regulates linoleic acid metabolism, DNA replication, phenylpropanoid biosynthesis, plant hormone signal transduction, and diterpenoid biosynthesis pathways in cucumber seedlings under low-temperature stress. By integrating the current KEGG pathway results with our previous research [16], we conclude that NO enhances chilling tolerance in cucumber seedlings by modulating GSH and flavonoid synthesis, as well as relative linoleic acid content. These findings align with earlier reports demonstrating that NO improves abiotic stress tolerance through the regulation of GSH [26], flavonoid synthesis [27], and increased saturation of fatty acids [16,28].

Phytohormones play an essential role in plant growth, development, and abiotic stress tolerance [29,30]. Hormones such as ETH, JA, ABA, SA, and GA regulate defense systems and enhance plant resilience to various stresses [19,31,32,33]. ETH is widely recognized as a fruit-ripening regulator. Ethylene response factors (ERFs) activate the transcription of ripening-related genes, ultimately promoting fruit ripening [34,35]. Moreover, studies have shown that ETH signaling regulates Cu/Zn-SOD and catalase activities, as well as photosystem II function, during cold acclimation [36]. Sougrakpam et al. reported that NO inhibits ethylene biosynthesis, thereby extending the post-harvest shelf life of agricultural produce [37]. In this study, genes involved in ETH signal transduction, including *ETR*, *MKK4_5*, *EIN3*, *EBF1_2*, and *ERF1*, were significantly upregulated under low-temperature stress in both cucumber cultivars, whereas *EIN2*, a negative regulator of ETH signaling, was downregulated. Under combined low-temperature and exogenous NO treatment, *EIN2* and two *ERF1* genes showed a non-significant upregulation trend (although not significant). These results suggest that NO may participate in the low-temperature response of cucumber by modulating ETH signaling.

SA is a key phytohormone involved in plant response to various stresses, including chilling stress [18]. Previous studies have indicated that SA can induce NO production [17]. In this study, low temperature regulated the expression of *NPR1*, *TGA,* and *PR1* in both cucumber cultivars. Furthermore, exogenous NO significantly upregulated *PR1* expression under low-temperature stress and counteracted the reduction in SA content caused by low temperature. These findings are consistent with the reported interactions between NO and SA signaling under abiotic stress [17,38].

JA and MeJA are recognized as naturally occurring phytohormones due to their widespread distribution and roles in various physiological processes [39]. NO serves as a signaling molecule that activates the MeJA-induced defense response and secondary metabolism activities in plant cells [40]. Our results demonstrated that low temperature promotes JA synthesis from alpha-linolenic acid by regulating *LOX2S*, *AOC*, *AOS*, and *OPR* gene expression, a process in which NO participates. Additionally, NO may contribute to JA-mediated low-temperature tolerance by modulating *MYC2* expression [16]. In support of this, Huang et al. also reported that NO induces JA production and activates JA-responsive gene expression [41].

The diterpenoid geranylegeranyl-PP is converted to GA9 by KAO, which is subsequently transformed into bioactive GA4 and GA1 via GA3ox. These active GAs participate in gibberellin signal transduction, while excess GA4 and GA1 can be degraded into their respective catabolites (GA34 and GA8) by GA2ox. Previous studies have shown that low temperature downregulates *SlGA2ox* expression, resulting in GA1 and GA4 accumulation and increased locule number in tomato [42]. Similarly, bioactive GA deficiency under low temperature impairs starch hydrolysis and sugar consumption, inhibiting rice seed germination [43], underscoring the importance of GAs in germination under cold stress. In this study, exogenous NO reversed the low-temperature-induced suppression of *KAO* and *GA3ox* and the upregulation of *GA2ox*, particularly in ‘Jinyou No. 1’. This suggests that NO may promote GA1 and GA4 accumulation under low temperature by enhancing *GA3ox* expression and repressing *GA2ox*, thereby improving low-temperature tolerance. Consistent with this, GA content increased under the combined NO and low-temperature treatment compared to low temperature alone. Furthermore, low temperature upregulated *GID1* but downregulated *DELLA* and *PIF4*, whereas NO restored *DELLA* and *PIF4* expression and suppressed the low-temperature induction of *GID1*. These results indicate that both GA biosynthesis and signaling are involved in the cucumber response to low temperature, with NO playing a regulatory role. In contrast to a reported study where GA signaling was suppressed under low temperature in rice seeds [43], our findings suggest that GA signaling acts positively, while ABA signaling acts negatively, in mediating cucumber cold response, implying an antagonistic interaction between GA and ABA pathways under low-temperature stress.

Among phytohormones, ABA remains one of the most extensively studied due to its physiological significance [44]. As an isoprenoid-class hormone, ABA plays a central role in regulating plant growth, development, and adaptation to environmental stresses. Its concentration often changes rapidly under adverse conditions, activating protective responses that enhance plant survival [45]. While abiotic stresses such as low temperature are generally known to induce ABA accumulation and improve stress tolerance [46], some studies report context-dependent responses. For instance, Gao-Takai et al. observed that high temperatures induced ABA accumulation and expression of *VlMybA1* transcription factors in their system [47]. In the present study, low temperature significantly downregulated genes involved in ABA synthesis and signaling, yet it also led to increased ABA content—a result consistent with certain earlier reports. Growing evidence indicates sophisticated crosstalk between NO and ABA signaling under stress conditions [48]. For example, NO-mediated activation of ABA biosynthesis has been shown to contribute to brassinosteroid (BR)-enhanced water stress tolerance in maize [30]. In our experiment, although not statistically significant, exogenous NO tended to upregulate ABA-related synthesis and signaling genes in cucumber under low-temperature stress. These observations suggest the involvement of NO in modulating ABA metabolism and signal transduction during cold stress.

## 4. Materials and Methods

### 4.1. Plant Materials and Low Temperature Treatment

Cucumber cultivars “Jinyan No. 4” and “Jinyou No. 1,” which differ in low-temperature tolerance, were obtained from the Xiangyun Seed Industry of Xintai in Shandong Province and the Department of Vegetables Research Institute in Tianjin, respectively. Seeds of both cultivars were soaked at 55 °C for 20 min, followed by 25 °C for 6 h, and then germinated on moist filter paper in the dark at 26 °C for 24 h. Uniformed germinated seeds were sown in plug trays containing a peat–vermiculite mixture (2:1, *v*/*v*). After full expansion of the cotyledons, the seedlings were transplanted into individual plastic pots (diameter × height: 120 × 110 mm) filled with the same substrate mixture. Seven days after transplantation, 50 mL of Hoagland’s nutrient solution was applied every four days. The seedlings were grown in an artificial climate chamber set at 25 °C/18 °C (day/night), with a 14 h/10 h photoperiod (light/dark), a light intensity of 300 μmol·m^−2^·s^−1^, and relative humidity of 75%/70% (light/dark). When the third true leaf was fully expanded, the seedlings of both cultivars were divided into three groups (30 seedlings in each group) and subjected to the following treatments: Yan_CK and You_CK, pretreated with distilled water once daily for two days, then maintained under normal conditions for 24 h; Yan_LT and You_LT, pretreated with distilled water once daily for two days, then exposed to low temperature (10 ± 1 °C day/6 ± 1 °C night, 14 h light/10 h dark, light intensity 100 μmol · m^−2^ · s^−1^) for 24 h; Yan_SNP and You_SNP, pretreated with 200 μmol·L^−1^ SNP (sodium nitroprusside, an NO donor) once daily for two days, then subjected to the same low-temperature conditions for 24 h. The third true leaf was sampled for all measurements. The experiment was arranged in a randomized design with three biological replicates.

### 4.2. RNA Extraction and Strand-Specific Library Construction

Fresh leaf samples (0.1 g) were collected from each biologicate of cucumber seedlings, flash-frozen in liquid nitrogen for 5 h, and stored at −80 °C until use. Total RNA was extracted using Trizol reagent according to the manufacturer’s instructions. RNA purity and integrity were assessed using a Nanodrop 2000 spectrophotometer (Thermo, Waltham, MA, USA) and 2% agarose gel electrophoresis, respectively. Accurate RNA quantification was performed using a Qubit fluorometer (Thermo, Waltham, MA, USA). The electrophoresis results are provided in Figure S1.

Strand-specific RNA libraries were constructed from quality-controlled mRNA samples as per the manufacturer’s protocol. Libraries from “Jinyan No. 4” seedlings subjected to the following treatments were designated as follows: Yan_CK (control), Yan_LT (low temperature: 10 ± 1 °C day/6 ± 1 °C night), and Yan_SNP (pretreated with SNP followed by low temperature). Similarly, libraries from “Jinyou No. 1” seedlings were labeled You_CK, You_LT, and You_SNP, corresponding to the same treatments. All six treatments were replicated three times in a completely randomized experimental design.

### 4.3. Transcriptome Sequencing and Quality Control

In this experiment, we sequenced a total of 18 samples representing six treatment groups (Yan_CK, Yan_LT, Yan_SNP, You_CK, You_LT, and You_SNP), each with three biological replicates. Following library preparation and quality control, transcriptome sequencing was performed on the HiSeq platform using a paired-end strategy with read lengths of 250–300 bp. Quality assessment of the raw reads was conducted with Fastqc (https://www.bioinformatics.babraham.ac.uk/projects/fastqc/, accessed on 15 April 2021), and low-quality as well as adaptor-containing sequences were removed [49]. High-quality reads were then mapped to the cucumber v3 reference genome, obtained from CuGenDB (http://cucurbitgenomics.org/, accessed on 15 April 2021), using Salmon [50].

### 4.4. DEG Identification and Enrichment Analysis

The clean data mapped to each gene were quantified and normalized to TPM (transcripts per kilobase of exon model per million mapped reads). Differential expression analysis was performed using DESeq2 [51], with genes satisfying |Log2 (fold change)| > 1 and an adjusted *p*-value < 0.01 defined as differentially expressed (Supplementary Data). These genes were subsequently subjected to functional enrichment analysis. Gene ontology (GO) and KEGG pathway annotations were retrieved from CuGenDB, and enrichment analysis was carried out using the clusterProfiler package in R (v 4.2.2) [52].

### 4.5. The Contents of Endogenous Hormones, Glutathione, Flavonoids, and Linoleic Acid Assays

The analysis of endogenous hormone contents was entrusted to Zhongke New Life Biotechnology Co., Ltd. (Shaoxing, China). and carried out using high-performance liquid chromatography (HPLC) and tandem mass spectrometry (MS) systems. For hormone quantification, 0.1 g of lyophilized tissue from the third true leaf of the cucumber plants was accurately weighed. A pre-chilled 80% methanol aqueous solution (10 mL in total: 5 mL, 3 mL, and 2 mL added sequentially) was used to grind the tissue into a homogenate under dim light in an ice bath. The extraction was performed in the dark at 4 °C for over 12 h with intermittent stirring (3–5 times). The extract was centrifuged at 5000 r/min for 10 min at 4 °C, and the supernatant was collected (the operation was repeated twice, and the supernatant was merged). Following extraction, the supernatant was vacuum freeze-concentrated to remove methanol, reconstituted in 8 mL of ammonium acetate (0.1 mol/L, pH = 9.0), and centrifuged at 15,000 r/min for 20 min. The supernatant was subsequently passed through a polyvinylpolypyrrolidone (PVPP) column and a diethylaminoethyl Sephadex (DEAE Sephadex A-25) column. Endogenous hormones were collected using a Sep-Pak C18 cartridge and then eluted with a 50% (*v*/*v*) methanol aqueous solution prior to HPLC-MS analysis.

The content of reduced glutathione (GSH) was determined following the method described by Nagalakshmi et al. [53]. Briefly, 0.3 g (fresh weight) of cucumber leaf tissue (the third true leaf) was homogenized in an ice bath with 3 mL of 5% sulfosalicylic acid and centrifuged at 12,000× *g* for 10 min. In total, 100 μL supernatant was mixed with 700 μL of 0.3 mM NADPH, 100 μL of 10 mM DTNB, and 150 μL of 125 mM NaPO_4_–6.3 mM EDTA buffer (pH 6.5). Then, 10 μL of glutathione reductase (50 U/mL) was added, and the change in absorbance at 412 nm was monitored. The GSH content was calculated according to the standard curve.

The flavonoid content was determined using a reagent kit (Nanjing Jiancheng Bioengineering Institute, China, A142-1-1). After being rinsed with distilled water and dried, the cucumber leaves were cut into small pieces. In total, 0.3 g of the tissue was ground into powder in liquid nitrogen. Then, 2 mL of extraction buffer was added, followed by extraction at 60 °C with shaking for 2 h. The mixture was centrifuged at 10,000× *g* for 10 min at room temperature. The supernatant was collected and used for flavonoid content determination according to the manufacturer’s instructions.

### 4.6. Quantitative Real-Time PCR (qRT-PCR) Analysis

We selected nine genes involved in phytohormone biosynthesis and plant hormone signal transduction pathways for qRT-PCR validation. Gene-specific primers were designed using the online tool Primer 3 (http://bioinfo.ut.ee/primer3-0.4.0/) and their specificity was verified by BLAST analysis against the cucumber genome in CuGenDB (http://www.cucurbitgenomics.org/). The cucumber *actin* gene served as the internal reference. All primer sequences are listed in Table S1. Quantitative real-time PCR was performed using the iCycler iQ Multicolor Real-time PCR Detection System (Bio-Rad, Hercules, CA, USA), and relative gene expression levels were calculated using the 2^−ΔΔCt^ method. The experiment included three biological replicates, each with three technical replicates.

## 5. Conclusions

**Figure 9:**
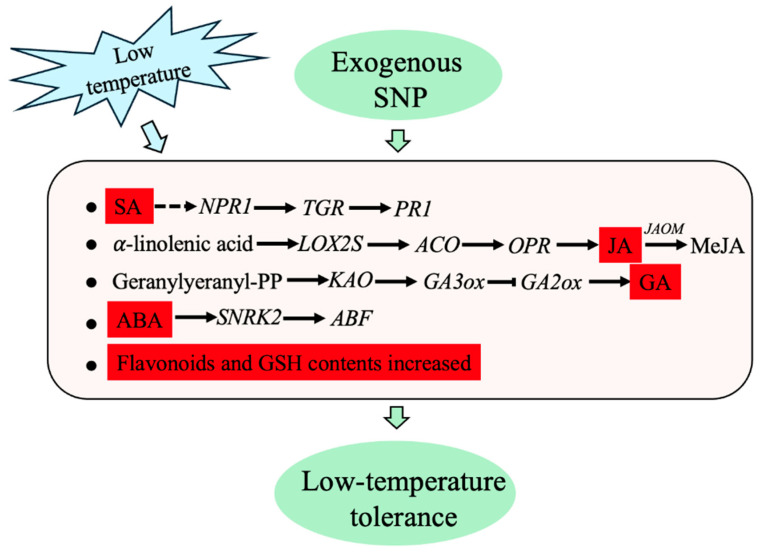
Schematic diagram of low-temperature tolerance in cucumber induced by exogenous SNP. The red box represents hormones or antioxidants.

Transcriptomics offers a powerful approach to elucidate the key molecular mechanisms underlying plant responses to environmental stress, thereby providing a foundation for the identification and validation of stress-related genes. This study provides insights into the mechanisms by which phytohormones (SA, JA, GA, ABA, and ETH), flavonoids, antioxidants (GSH), and linoleic acid contribute to low-temperature stress tolerance in cucumber seedlings, through integrated transcriptomic and physiological analyses (Figure 9). Exogenous NO was found to participate in the cucumber response to low temperature by regulating the synthesis of active GA from diterpenoids, the production of MeJA and linoleic acid from α-linolenic acid, and the signal transduction pathways of ABA, GA, JA, SA, and ETH. Although the regulatory interplay between NO and phytohormones under low-temperature stress has not been fully elucidated, this study offers new perspectives on enhancing plant cold resistance through exogenous SNP application and its interaction with endogenous hormones. Further research is needed to elucidate the molecular mechanisms underlying NO-induced hormone signaling in enhancing cucumber low-temperature tolerance.

## Data Availability

The original contributions presented in this study are included in the article. Further inquiries can be directed to the corresponding author(s).

## Supplementary Materials

The following supporting information can be downloaded at https://www.mdpi.com/article/10.3390/plants14213275/s1: Table S1: Quantitative real-time PCR primer sequences; Table S2: Data description of RNA-Seq reads for the six cucumber samples with three replicates; Figure S1: RNA electrophoresis image. 1–3 represent Yan_CK; 4–6 represent Yan_LT; 7–9 represent Yan_SNP; 10–12 represent You_CK; 13–15 represent You_LT; 16–18 represent You_SNP; Figure S2: Comparison rate between clean readings of each sample and annotated genome (A); The mapped rations of each sample (B); Figure S3: The numbers of differently expressed genes (DEGs) in the different treatments. The treatment groups were defined as follows: Yan_CK and You_CK: the seedlings of ‘Jinyan No 4.’ and Jinyou No. 1 were pre-treated with distilled water once daily for two days and then grown under normal conditions for 24 h, respectively; Yan_LT and You_LT: the seedlings of ‘Jinyan No 4.’ and Jinyou No. 1 were pre-treated similarly with distilled water but then exposed to low temperature for 24 h, respectively; Yan_SNP and You_SNP: the seedlings of ‘Jinyan No 4.’ and Jinyou No. 1 were pre-treated with exogenous SNP (200 μmol · L^−1^) once daily for two days, then grown under low temperature for 24 h, respectively; Figure S4: GO enrichment analysis of differentially expressed genes (DEGs) for Yan_LT vs. Yan_CK (A), Yan_SNP vs. Yan_LT (B), You_LT vs. You_CK (C), and You_SNP vs. You_LT (D). The treatment groups were defined as follows: Yan_CK and You_CK: the seedlings of ‘Jinyan No 4.’ and Jinyou No. 1 were pre-treated with distilled water once daily for two days and then grown under normal conditions for 24 h, respectively; Yan_LT and You_LT: the seedlings of ‘Jinyan No 4.’ and Jinyou No. 1 were pre-treated similarly with distilled water but then exposed to low temperature for 24 h, respectively; Yan_SNP and You_SNP: the seedlings of ‘Jinyan No 4.’ and Jinyou No. 1 were pre-treated with exogenous SNP (200 μmol · L^−1^) once daily for two days, then grown under low temperature for 24 h, respectively. “*” represents significantly enriched; Figure S5: KEGG enrichment analysis of DEGs for Yan_LT vs. Yan_CK (A), Yan_SNP vs. Yan_LT (B), You_LT vs. You_CK (C), and You_SNP vs. You_LT (D). The treatment groups were defined as follows: Yan_CK and You_CK: the seedlings of ‘Jinyan No 4.’ and Jinyou No. 1 were pre-treated with distilled water once daily for two days and then grown under normal conditions for 24 h, respectively; Yan_LT and You_LT: the seedlings of ‘Jinyan No 4.’ and Jinyou No. 1 were pre-treated similarly with distilled water but then exposed to low temperature for 24 h, respectively; Yan_SNP and You_SNP: the seedlings of ‘Jinyan No 4.’ and Jinyou No. 1 were pre-treated with exogenous SNP (200 μmol · L^−1^) once daily for two days, then grown under low temperature for 24 h, respectively.
