# Supplementary material for: Phytohormone Response to Exogenous Nitric Oxide in Cucumber Under Low-Temperature Stress
Source: Plants (Basel). 2025 Oct 27;14(21):3275. doi: 10.3390/plants14213275 (PMC12610254; doi:10.3390/plants14213275)
Supplement: Supplementary file 1 [file plants-14-03275-s001.zip › plants-3870869-supplementary.pdf]

Supplementary Figures and Tables

Table S1. Quantitative real-time PCR primer sequences.

name	Unigene ID	Forward primer (5'-3')	Reverse primer (5'-3')
Actin	***CsaV3_6G041900.1***	**GTGCCTGCTATGTATGTTG**	**CTCCGATGGTGATGACTT**
JAOM	***CsaV3_3G045390.1***	**ATTTGGGCTGCTCATCTGG**	**TGAACTTCGGGTAGTGGGT**
LOX2S	***CsaV3_4G023930.1***	**GGCCCTCTCAATTTCGTCT**	**TAGGAGTGTTGGATGGCAA**
AOC	***CsaV3_5G023060.1***	**GGCATCTTTGAAGGCGTTT**	**CCGCGATCCCCTTCAAATA**
MYC2	***CsaV3_3G001710.1***	**AAGAAGAATCATCCGGCGG**	**CCCCATCTTCACTGTTGCT**
PR1	***CsaV3_7G007620.1***	**GACCTGTCAGTTGGGATGA**	**CCATATGGCCCGTTAGAGT**
KAO	***CsaV3_6G006520.1***	**GTGATCCCCTTCCTCCTCT**	**AAGCGTTTCGAACCCAACT**
GA3oX	***CsaV3_2G031670.1***	**GGGGACTTGTTTCACATCC**	**GGCGATACACAGAAATCCG**
GA2oX	***CsaV3_4G007790.1***	**GGGCTGGGTCGAATACATT**	**ACGCCATATTCCTCACAGC**
ERF1	***CsaV3_3G012170.1***	**CTAATCTCCGAGGGCACAT**	**GCGGCGAACTCCTCTATAA**

Table S2. Data description of RNA-Seq reads for the six cucumber samples with three replicates

samples	Total reads (M)	Total bases (G)	Mapping ratio	Clean total reads (M)	Clean total bases (G)	Q30 bases
Yan_CK1	48.72	7.31	71.67	48.56	7.25	0.93
Yan_CK2	45.28	6.79	70.85	45.14	6.75	0.93
Yan_CK3	53.18	7.89	68.88	53	7.92	0.93
Yan_LT1	42.68	6.4	61.18	42.51	6.36	0.93
Yan _LT2	52.75	7.91	65.75	52.59	7.86	0.93
Yan_ LT3	51.94	7.79	66.06	51.74	7.73	0.93
Yan_SNP1	51.02	7.65	63.85	50.84	7.6	0.93
Yan_SNP2	55.94	8.39	68.41	55.74	8.34	0.93
Yan_SNP3	52.85	7.93	68.35	52.72	7.88	0.94
You_CK1	49.76	7.46	62.12	49.54	7.41	0.92
You_CK2	50.95	7.64	63.62	50.77	7.6	0.93
You_CK3	48.14	7.22	63.34	47.97	7.17	0.92
You_ LT1	53.28	7.99	67.35	53.14	7.94	0.94
You_ LT2	49.65	7.45	68	49.46	7.39	0.92
You_ LT3	52.65	7.9	68.07	52.49	7.84	0.93
You_SNP1	58.16	8.72	66.31	57.94	8.66	0.93
You_SNP2	52.89	7.93	66.07	52.73	7.88	0.93
You_SNP3	45.35	6.8	66.32	45.17	7	0.92

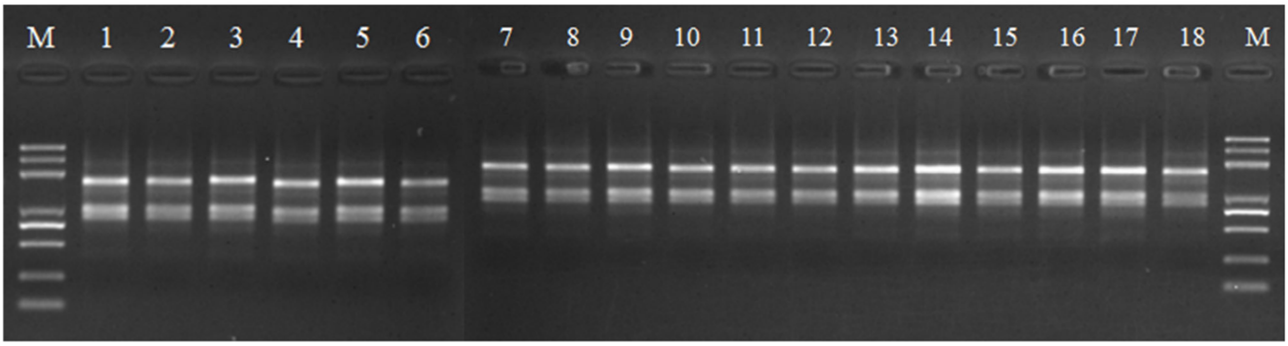

Figure S1. RNA electrophoresis image. 1-3 represent Yan_CK; 4-6 represent Yan_LT; 7-9 represent Yan_SNP; 10-12 represent You_CK; 13-15 represent You_LT; 16-18 represent You_SNP.

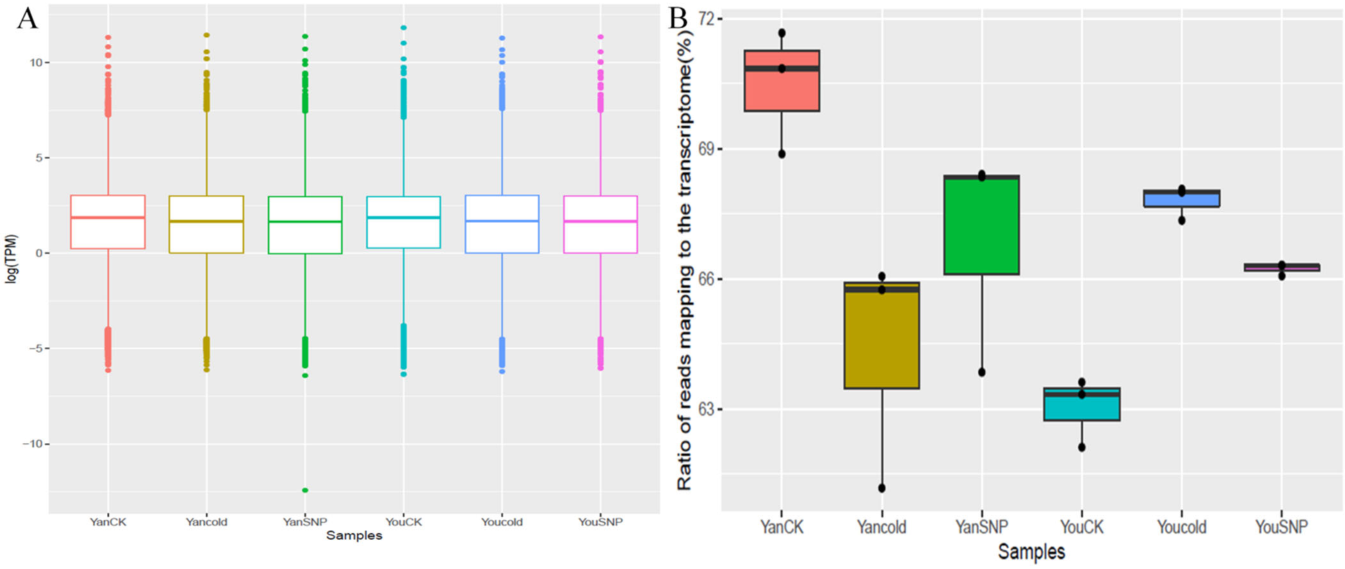

Figure S2. Comparison rate between clean readings of each sample and annotated genome (A); The mapped rations of each sample (B).

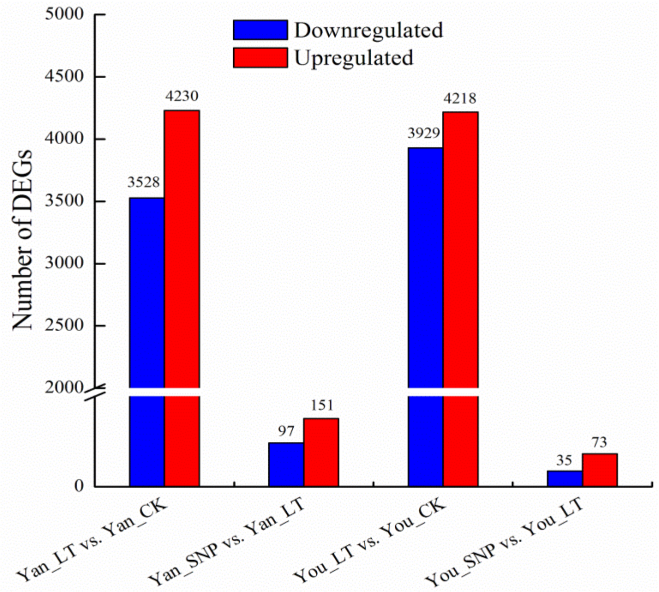

Figure S3. The numbers of differently expressed genes (DEGs) in the different treatments. The treatment groups were defined as follows: Yan_CK and You_CK: the seedlings of 'Jinyan No 4.' and Jinyou No. 1 were pre-treated with distilled water once daily for two days and then grown under normal conditions for 24 h, respectively; Yan_LT and You_LT: the seedlings of 'Jinyan No 4.' and Jinyou No. 1 were pre-treated similarly with distilled water but then exposed to low temperature for 24 h, respectively; Yan_SNP and You_SNP: the seedlings of 'Jinyan No 4.' and Jinyou No. 1 were pre-treated with exogenous SNP (200 µmol · L^{-1}) once daily for two days, then grown under low temperature for 24 h, respectively.

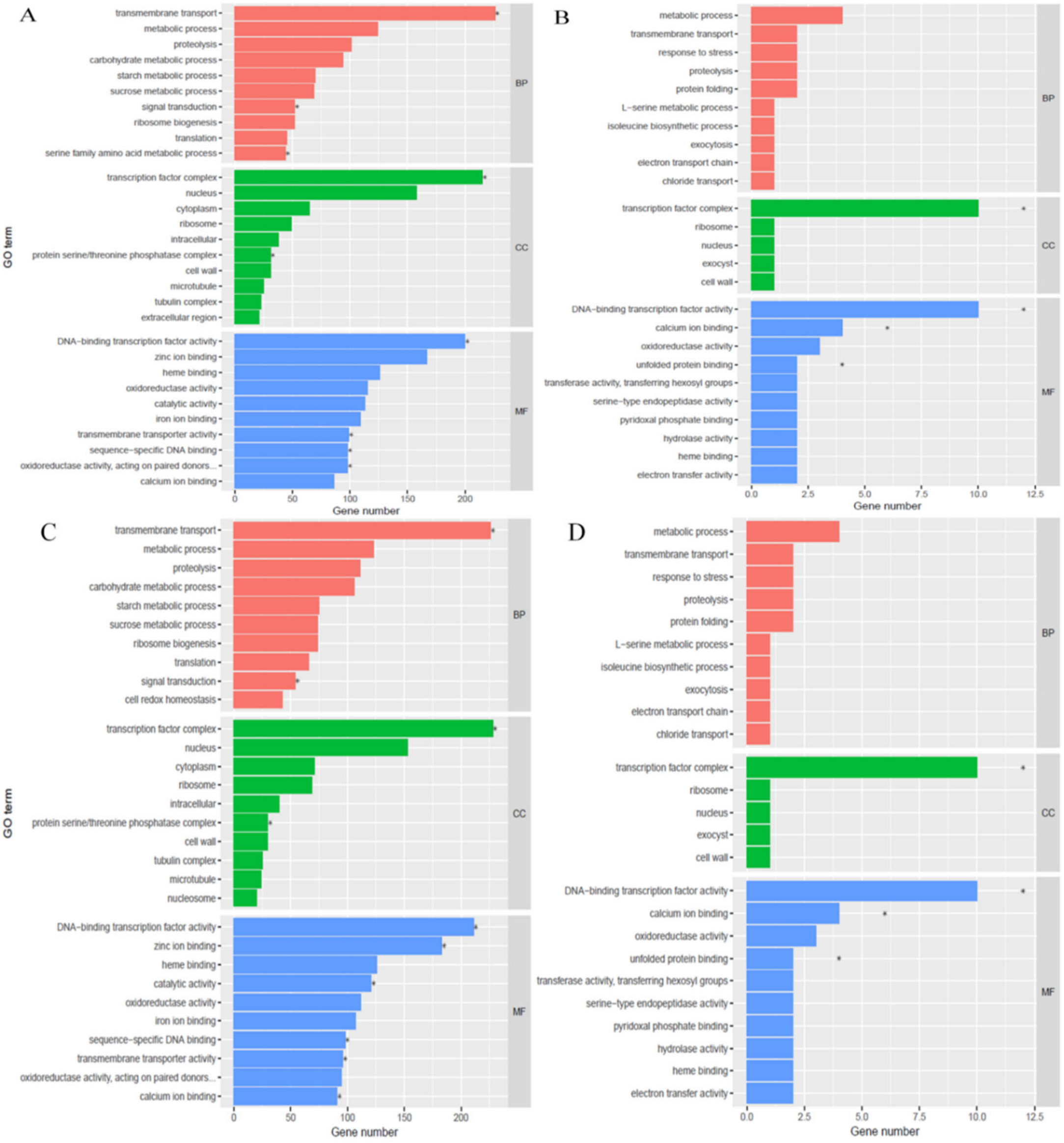

Figure S4. GO enrichment analysis of differentially expressed genes (DEGs) for Yan_LT vs. Yan_CK (A), Yan_SNP vs. Yan_LT (B), You_LT vs. You_CK (C), and You_SNP vs. You_LT (D). The treatment groups were defined as follows: Yan_CK and You_CK: the seedlings of 'Jinyan No 4.' and Jinyou No. 1 were pre-treated with distilled water once daily for two days and then grown under normal conditions for 24 h, respectively; Yan_LT and You_LT: the seedlings of 'Jinyan No 4.' and Jinyou No. 1 were pre-treated similarly with distilled water but then exposed to low temperature for 24 h, respectively; Yan_SNP and You_SNP: the seedlings of 'Jinyan No 4.' and Jinyou No. 1 were pre-treated with exogenous SNP (200 μmol · L^{-1}) once daily for two days, then grown under low temperature for 24 h, respectively. "*" represents significantly enriched.

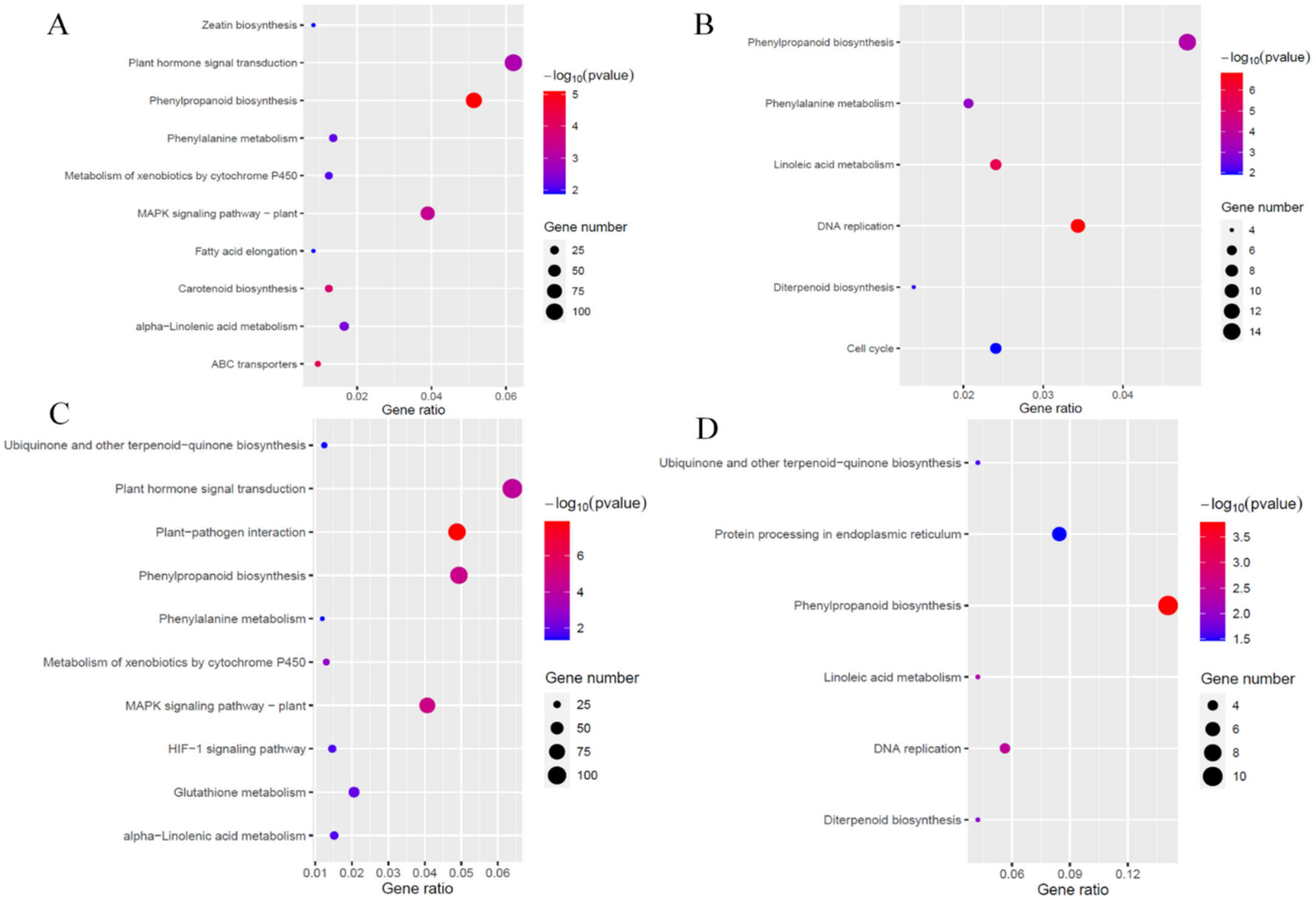

Figure S5. KEGG enrichment analysis of DEGs for Yan_LT vs. Yan_CK (A), Yan_SNP vs. Yan_LT (B), You_LT vs. You_CK (C), and You_SNP vs. You_LT (D). The treatment groups were defined as follows: Yan_CK and You_CK: the seedlings of 'Jinyan No 4.' and Jinyou No. 1 were pre-treated with distilled water once daily for two days and then grown under normal conditions for 24 h, respectively; Yan_LT and You_LT: the seedlings of 'Jinyan No 4.' and Jinyou No. 1 were pre-treated similarly with distilled water but then exposed to low temperature for 24 h, respectively; Yan_SNP and You_SNP: the seedlings of 'Jinyan No 4.' and Jinyou No. 1 were pre-treated with exogenous SNP (200 μmol · L^{-1}) once daily for two days, then grown under low temperature for 24 h, respectively.
